# Supplementary material for: Interpreting blood GLUcose data with R package iglu
Source: PLoS One. 2021 Apr 1;16(4):e0248560. doi: 10.1371/journal.pone.0248560 (PMC8016265; doi:10.1371/journal.pone.0248560)
Supplement: Attachment — Submitted filename: ResponseReviews.pdf [file pone.0248560.s001.pdf]

Response to Reviews for "Interpreting blood GLUcose data with R package iglu"

Thank you for the opportunity to revise our manuscript. We are grateful to the Editor and the reviewers for the comments and suggestions that helped significantly improve both the manuscript and the software. Our revisions are detailed below. We use *cursive font* for your comments, and standard font for our response.

R.1 Editor's Comments

1. *Please perform a careful copy edit as there are typos and missing references.*

   Thank you for pointing this out. We have performed additional proofreading and editing to correct the typos.

2. *In fig 2c, if these are 24 hour averages how can the x-axis unit be hours?*

   We apologize for the confusion. What we meant is the following: for each subject, the daily profiles are averaged across the same time points, and the plot displays these averaged glucose values from midnight to midnight. We realize now that our default title for this type of plot was confusing, so we have changed it from "24 hour averages for all subjects" to "Average glucose values for all subjects across days", and modified Figure 2 accordingly.

3. *The Shiny interface is an extremely useful step, reducing the need for the user to have programming experience. However, the user still needs to get the data into 3 column format, which is not the format exported by the CGM software. Therefore, this step may require programming or manual editing of the data, which could introduce error.*

   This is an excellent point. Our rationale for using .csv is that our original aim in developing iglu was to assist researchers with the analyses of multi-subject CGM data that often arise as a result of clinical trials, rather than individual subject data. In our experience, multi-subject CGM data is typically already processed by the corresponding data curation team from the original meter-specific format. For example, the data for this study (`https://doi.org/10.1371/journal.pbio.2005143`) is available as a supplement in GZ format, whereas the data for this study (`https://zenodo.org/record/1421616#.YAnk7C2ZNm8`) when extracted from .tgz has individual .csv files for each of the 9 subjects. In fact, based on our experience collecting publicly available CGM data (`https://github.com/irinagain/Awesome-CGM`), most of such data is stored as .csv. We do, however, completely agree that adding more data formats, and in particular meter-specific formats, will strengthen the package and the Shiny App, and is definitely a current limitation in functionality. Some existing R packages for CGM have such functionality (e.g. cgmanalysis), but quite a substantial effort is required for seamless integration with Shiny interface, especially since the proprietary formats change from one meter to another, and new CGM meters continue to be developed. We do, however, hope

to be able to address this limitation in future work, and acknowledge this limitation in the conclusion:

> There are several limitations to our comparison of iglu with existing CGM software. First, the R interface assumes that the CGM data is already loaded into R as a data frame, which requires users to have sufficient R knowledge for data processing. The Shiny app currently only allows to load CGM data in .csv format, and thus it also requires initial pre-processing by the user, albeit not necessarily in R. This is not the case for CGManalyzer or cgmanalysis, which can work directly with specialized data formats from many popular CGMs. Continuous development of new CGM meters coupled with varying data formats across meters present definite challenges for any CGM software. We hope to contribute to addressing these challenges by leveraging complimentary functionality of existing open-source CGM software and our own updates to iglu in the future.

R.2 Response to Reviewer 1

The authors present the R package "iglu" and accompanying Shiny app for visualization and analysis of continuous glucose monitor (CGM) data. The software calculates CGM metrics and generates figures not available in other packages, and is accessible to users with little programming experience. It is an excellent piece of software that will significantly ease the burden of analysis for many researchers.

Thank you for the positive feedback.

Minor revisions for the paper:

1. *There are a couple of small typos, so the paper could do with one more round of copy editing*

   Thank you for pointing this out. We have performed additional proofreading and editing to correct the typos.

2. *In the interest of fairness, it would be worth including in Table 2 metrics calculated by CGManalyzer and cgmanalysis that are not included in iglu.*

   Thank you for your suggestion. We agree, and we have expanded Table 2 accordingly based on the documentation provided for each software package.

   The summary of main changes in Table 2 compared to the previous version is as follows:

   - Added "Multiscale entropy" which is implemented in CGManalyzer, but not in cgmanalysis or iglu
   - Added newly implemented in iglu COGI and MAG metrics, their implementation has been suggested by another reviewer
   - Added "Time in range" separately from "Percent in range"
   - Added "Excursions count" which is implemented in cgmanalysis but not in CGManalyzer or iglu
   - Added "Day/night metrics (SD, min, max, AUC)" which are implemented in cgmanalysis but not in CGManalyzer or iglu

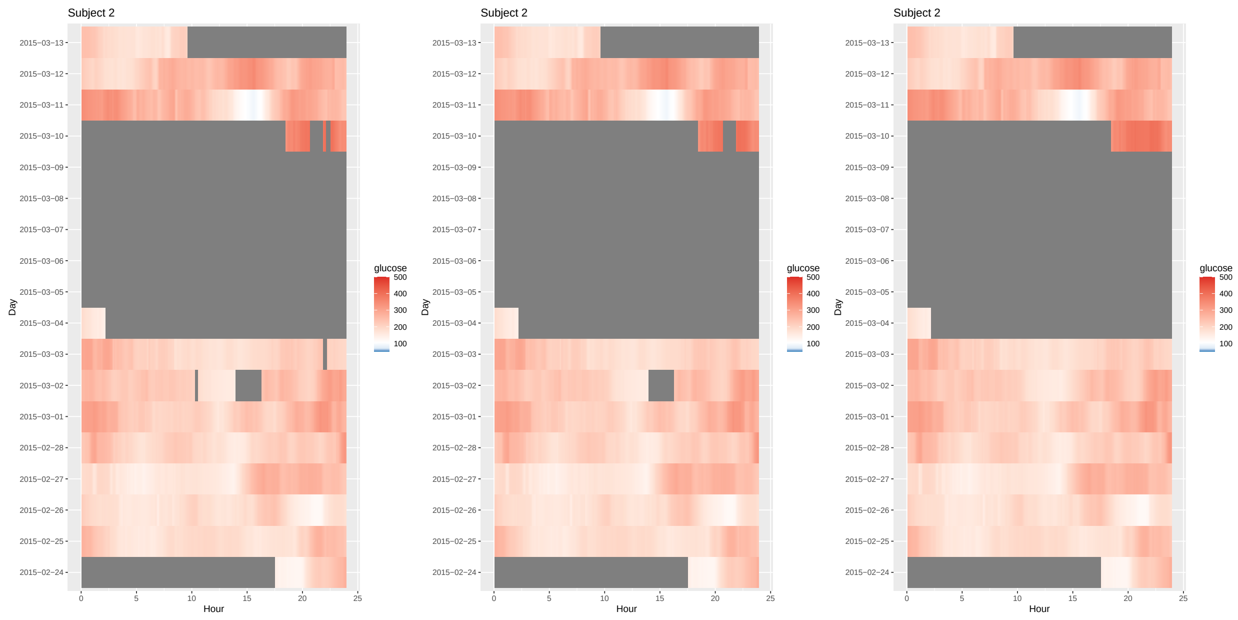

Figure R.1: Unsorted lasagna plots for Subject 2 using different values of the inter_gap parameter for linear interpolation in 'blue-red' color scheme: from left to right the values of 10 min, 45 min (default), and 150 min (2 hours, 30 min) are used, correspondingly.

To simplify the comparisons and to follow your suggestion, we have only focused our comparison on various metrics rather than other functionality (e.g. visualization capabilities or the type of accepted data). We have, however, added some discussion of limitations of our package in that regard to the Conclusions section (see also our response to your point 6 below).

3. *Please add a little bit more detail on how the package handles missing values. Does the CGMS2DayByDay() function automatically fill in missing data using linear interpolation? Or does it fill in with NA values? Also, the caption of Figure 1 claims that metric calculations are not affected by missing glucose values, but this cannot be true for all metrics so clarification is needed.*

   Thank you for your comments. The CGMS2DayByDay() function only fills in missing values that are less than inter_gap minutes apart, the values that are more than inter_gap minutes apart are replaced with NAs. By default, the function uses inter_gap = 45 minutes, however this value can be adjusted by the user. Our rationale for the inter_gap parameter is as follows. Suppose the meter frequency is 5 minutes, and we observe values 100, NA, 150 at time points 0, 5 and 10 minutes. Given that glucose values cannot biologically change too abruptly, we believe it is reasonable in this case to interpolate the NA value at 5 min, and replace that NA value with 125. On the other hand, for Subject 2 (see Figure R.2) there is a large gap of measurements due to missing values for several days, and we believe it's impossible to accurately impute those values. Thus, applying CGMS2DayByDay() function to Subject 2 data will leave that large gap as NA. To better illustrate the effect of the inter_gap parameter, Figure R.1 shows lasagna plots for Subject 2 with various values for inter_gap. Observe that the large interval of missing data consistently remains NA, whereas the smaller intervals get filled in as the value of inter_gap grows.

We have added additional clarifications on CGMS2DayByDay() in the manuscript which now reads as follows:

> The calculations of these variability metrics require evenly spaced glucose measurements across time; however, this is not always the case in practice due to missing values and misalignment of CGM measurement times across subjects (e.g. measurement at 17:30 for Subject 1, but at 17:31 for Subject 2). In order to create a uniform evenly spaced grid of glucose measurements, iglu provides the function CGMS2DayByDay. This function is automatically called for metrics requiring the evenly spaced grid across days, however the user can also access the function directly. The function works on a single subject's data, and has three outputs.

```
str(CGMS2DayByDay(example_data_1_subject))
List of 3
  $ gd2d        : num [1:14, 1:288] NA 112.2 92 90.1 143.1 ...
  $ actual_dates: Date[1:14], format: "2015-06-06" "2015-06-07" ...
  $ dt0         : num 5
```

> The first part of the output, gd2d, is the interpolated grid of values. Each row corresponds to one day of measurements, and the columns correspond to an equi-distant time grid covering a 24 hour time span. The grid is chosen to match the frequency of the sensor (5 minutes in this example leading to $(24 * 60)/5 = 288$ columns), which is returned as dt0. The linear interpolation is only performed between observed CGM values that are less than inter_gap minutes apart, otherwise missing values are inserted. By default, the function uses inter_gap = 45 minutes, however this value can be adjusted by the user. The returned actual_dates allows one to map the rows in gd2d back to the original dates. The achieved alignment of glucose measurement times across the days enables both the calculation of corresponding metrics, and the creation of lasagna plots discussed in the next section.

Regarding your point on missing values affecting metrics calculations, we have revised Figure 1 following your suggestions below, and eliminated confusing language. What we meant is that the previously present linear interpolation artifact on Figure 1 did not represent glucose values that were used for calculations. Now that the artifact is no longer present, Figure 1 provides an accurate representation of the data that is actually being used. Furthermore, most metrics do not use linear interpolation/imputation via CGMS2DayByDay() at all. For example, any time-independent metric from Table 1 is calculated solely based on all the available not NA measurements for the given subject. The active_percent() function can be used to validate % of time the CGM is active (proportion of not NA values) within the observed period, and is also acting on the original (not interpolated) measurements. Secondly, even when the linear interpolation is necessary via CGMS2DayByDay() (for example, to compute average across days at exactly the same time point), this linear interpolation is only performed within the inter_gap interval (see Figure R.1), missing values are inserted between any two measurements that are more than inter_gap minutes apart. Thus, the functions in iglu allow one to work with missing data with reasonable safeguards against "dangerous" imputation, and provide the user with the ability to assess the reliability of estimates due to missing data via the active_percent. This last point is now reflected in the manuscript as follows:

> Finally, iglu also allows one to assess the reliability of estimated CGM metrics by providing information on the number of days of data collection together with %

of time the CGM device was active during those days (% of non-missing measurements). This information is automatically provided as part of the standardized AGP output discussed in the next section, and can also be obtained directly by calling the function active_percent.

```
active_percent(example_data_5_subject)
# A tibble: 5 x 5
  id        active_percent ndays     start_date          end_date
  <fct>              <dbl> <drtn>    <dttm>              <dttm>
1 Subject 1           79.8 12.7 days 2015-06-06 16:50:27 2015-06-19 08:59:36
2 Subject 2           58.9 16.7 days 2015-02-24 17:31:29 2015-03-13 09:38:01
3 Subject 3           92.1  5.8 days 2015-03-10 15:36:26 2015-03-16 10:11:05
4 Subject 4           98.7 12.9 days 2015-03-13 12:44:09 2015-03-26 10:01:58
5 Subject 5           95.8 10.6 days 2015-02-28 17:40:06 2015-03-11 08:04:28
```

According to [17] (Riddlesworth et al., 2018), 10-14 days of CGM measurements are generally sufficient for assessing outcomes in clinical trials, and for determining potential adjustments to diabetes management based on retrospective review. Given these recommendations, the estimates of CGM parameters for Subject 3 are less reliable than the estimates for other subjects in the example dataset.

4. *Figure 4 and the section on "relationship between metrics," while interesting, seem beyond the scope of this manuscript. The hierarchical-clustering tool does not appear to be available in the Shiny app (although adding it could be extremely useful), which makes the inclusion of this clustered heatmap slightly confusing.*

   Thank you for your comment. The general comparison across metrics is beyond the scope of this manuscript. However, in the process of writing this manuscript, we realized that the readers who are new to CGM literature (in particular, statisticians who are new to this type of data) may feel overwhelmed by the sheer amount of available metrics and confused by their names, and thus may benefit from at least some guidance on the metrics concordance with each other. So our goal here is a simple illustration of metrics "closeness" on the example dataset, and we believe it is a very useful starting point for such new users.

   We indeed have not added hierarchical-clustering tool to the Shiny app as it's part of another R package (pheatmap) rather than iglu itself, and is in general not specifically designed for CGM metrics or CGM data. We are, however, inspired by your comments, and as a result would like to further investigate potential usefulness of metrics hierarchical clustering for CGM data interpretation and analysis. We believe such investigation will require a larger scale application of the approach (e.g. using data from subjects with both Type I and Type II diabetes across several CGM meters with larger sample sizes), and we hope to pursue it in future work.

5. *Please add some brief comparisons to demonstrate how closely this software agrees with other packages. This does not need to be an in-depth statistical analysis, as that would also be beyond the scope of the paper. However, the primary concern for many readers will be the accuracy of the calculations, so brief side-by-side comparisons between packages would be reassuring.*

   We agree that it would be good to have a close comparison with other packages; however, we argue that this is a very substantial and difficult undertaking on a large scale (for all metrics) given the differences in required file formats, differences in default values used for some metrics and differences in algorithms used for metrics calculation. Furthermore, comparison with other software does not necessarily represent a comparison with gold standard. In fact, we

believe it will be of great value to the community if a separate work is conducted on this very topic by providing a gold standard using publicly available CGM data against which various packages and algorithms can be compared (public data will make it easier for others to attest to those standards). Some of the difficulties associated with testing metric values across packages are also summarised in Vigers et al. (2019).

On our end, each function was tested by at least two different people from the team to make sure that the implementation agrees with the prescribed formulas of each respective algorithm, and in fact, we were even able to identify some typos in some published formulas early on in this process that were reported here:

- Gaynanova, I., Urbanek, J., Punjabi, N. M. (2018). Corrections of Equations on Glycemic Variability and Quality of Glycemic Control. Diabetes technology therapeutics, 20(4), 317-317.

Following your suggestion, we also used the example data from 5 subjects to cross-compare some selected metrics across the packages (we selected the most common metrics for which either there are no parameters, or we are confident in agreement of both the underlying parameters, and the utilized algorithm). We found that Summary Statistics (min, max, mean, quantiles) and overall SD are in perfect agreement between iglu, CGManalyzer and cgmanalysis for all 5 subjects. Additionally, we found that iglu and cgmanalysis have perfect concordance in the values of GMI, eA1C, CV, % of glucose values in various ranges and J-index. There is a slight (less than 1%) disagreement in % of time CGM is active between iglu and cgmanalysis, which we suspect is due to different rounding schemes being used. There is also a slight disagreement in CONGA values (using 1 hour) between all CGManalyzer, cgmanalysis and iglu which we believe is likely due to differences in treatment of missing values and interpolation schemes, however the disagreement is quite minor. The selected comparisons are in Table R.1, which is now included in the main manuscript with brief summary.

We also outlined the aforementioned limitations of our comparison in the Conclusion section (see also our response to your point 6).

6. *Please add some limitations to the conclusion section.*

   We agree that there are limitations to our software, and we have added the following paragraph to the conclusion:

   > There are several limitations to our comparison of iglu with existing CGM software. First, the R interface assumes that the CGM data is already loaded into R as a data frame, which requires users to have sufficient R knowledge for data processing. The Shiny app currently only allows one to load CGM data in .csv format, and thus it also requires initial pre-processing by the user, albeit not necessarily in R. This is not the case for CGManalyzer or cgmanalysis, which can work directly with specialized data formats from many popular CGMs. Nevertheless, continuous development of new CGM meters coupled with varying data formats across meters present definite challenges for any CGM software. Secondly, while the list of metrics implemented in iglu is more comprehensive compared to other R packages on CGM (Table 2), it still lacks some functionality that may be desired as part of the AGP output (Danne et al. 2017), specifically the count of hypoglycemia/hyperglycemia excursions, and separation of metrics into sleep/wake time periods. Thirdly, while the agreement of metrics values across software packages is encouraging, it does

Table R.1: Comparison of selected metrics across R packages using example data

Metric name	Subject id	CGManalyzer	cgmanalysis	iglu
Mean	Subject 1	123.7	123.7	123.7
	Subject 2	218.5	218.5	218.5
	Subject 3	154.0	154.0	154.0
	Subject 4	129.7	129.7	129.7
	Subject 5	174.6	174.6	174.6
SD	Subject 1	33.3	33.3	33.3
	Subject 2	52.4	52.4	52.4
	Subject 3	44.8	44.8	44.8
	Subject 4	29.1	29.1	29.1
	Subject 5	55.6	55.6	55.6
% CGM is Active	Subject 1	×	79.0	79.8
	Subject 2	×	58.0	58.9
	Subject 3	×	92.0	92.1
	Subject 4	×	98.0	98.7
	Subject 5	×	95.0	95.8
GMI	Subject 1	×	6.3	6.3
	Subject 2	×	8.5	8.5
	Subject 3	×	7.0	7.0
	Subject 4	×	6.4	6.4
	Subject 5	×	7.5	7.5
J-index	Subject 1	×	24.6	24.6
	Subject 2	×	73.3	73.4
	Subject 3	×	39.5	39.5
	Subject 4	×	25.2	25.2
	Subject 5	×	54.4	54.4
CONGA ($n = 1$ hour)	Subject 1	24.7	25.7	25.9
	Subject 2	19.9	25.1	25.7
	Subject 3	38.2	41.0	39.5
	Subject 4	23.2	22.6	23.3
	Subject 5	49.0	50.0	49.3

not necessarily signify the agreement with gold standard (see also the discussion in [4](Vigers et al., 2019)). Furthermore, a comprehensive cross-comparison across packages is quite difficult as it requires a careful adjustment for potential differences in default parameters used in metrics calculations, in handling of missing values, and in underlying algorithms used. However, we believe that the explicit metric values provided in Table R.1 coupled with public availability of our example dataset will serve as a useful preliminary step towards this endeavor. We hope to address some of these limitations in the future by leveraging complimentary existing open-source CGM software and our own updates to iglu.

Minor suggestions for the software:

1. *Although the graphing artifact (Figure 1, subject 2) is obvious in this example data, smaller artifacts may be less obvious in real-life data. Missing values should be blank space in the plots to avoid confusion.*

   Thank you for your comments. We followed the suggestion and modified the plot_glu function to prevent automatic linear interpolation in the plots over the values that are more than

inter_gap minutes apart (with the default inter_gap = 45 minutes). Figure R.2 shows updated Figure 1 in the manuscript. Note that we also have changed the default target range to [70, 180] mg/dL as has been suggested by another reviewer.

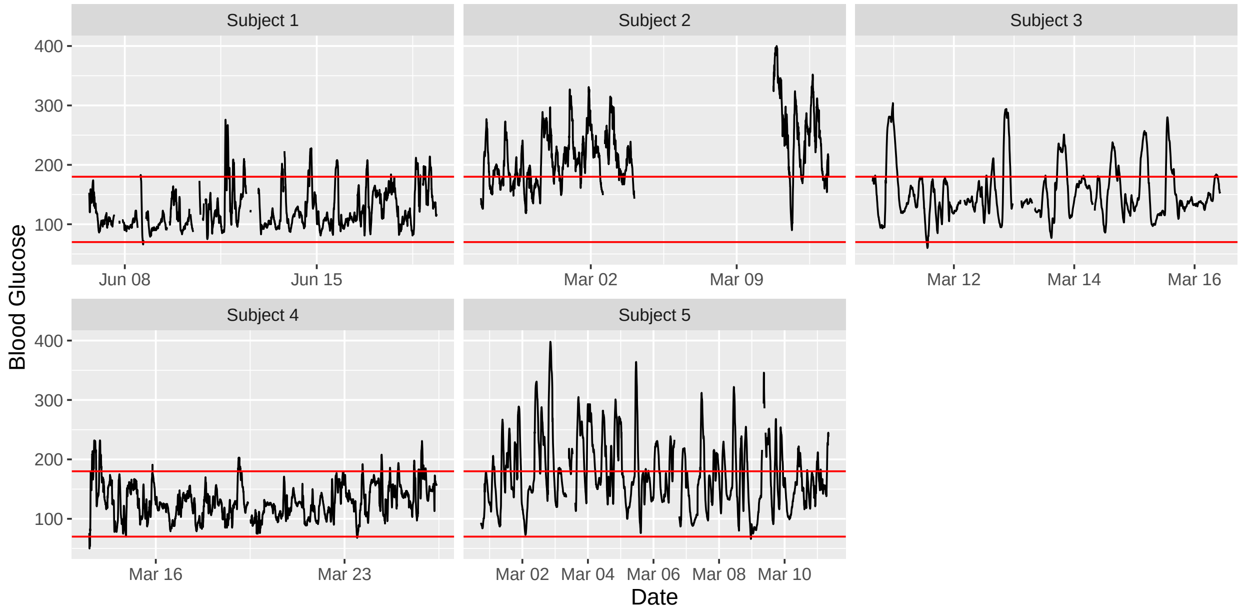

Figure R.2: Time series plots for five subjects. Selected target range is [70, 180] mg/dL.

2. *After loading the 5 subject example data in R, writing to .csv, and loading into the Shiny app, the active_percent() function produces Error: unused arguments (180, 200, 250).*

   Thank you for alerting us to this bug in the Shiny app which we have missed. It has been fixed.

3. *The visualizations are generally excellent as they are, but one suggestion would be to look into making the plots interactive using the plotly graphing library in R. This is not necessary for publication, but perhaps a recommendation for future updates.*

   Thank you very much for the great suggestion. We agree that it would be nice to have interactive plots. One design challenge, however, is that the interactive plots cannot be easily exported (our current static plots could be exported with one click into pdf, png or eps from the Shiny App). So ideally, we would like the users to have the ability to both export the plots, and be able to interact with the plots, and in future updates we hope to find a solution that will allow both.

4. *Many researchers use packages like this to generate metrics for further analysis, so it might be nice to have the option to download all the metrics at once rather than manually combining results from multiple functions. Again, this is not a necessary change for publication, but simply a recommendation for the future.*

   Thank you for your suggestion. We have added a new function called all_metrics that implements all current metrics in the package using their default parameter values. The functionality can also be accessed from the Shiny App by scrolling down to the end of Metrics List for "All Metrics".

R.3 Response to Reviewer 2

1. *The paper is well written. This work goes beyond previous methods/software for analysis of CGM data. There is a need for such open source software. This software includes several metrics that were not included in previous software. The work is done well technically.*

   Thank you for the positive feedback.

2. *The introduction of Lasagna plots is new and novel. That approach is closely related to the use of stacked bar charts as introduced by Rodbard in 2009, and now included in nearly all outputs for CGM data. It remains to be see to what extent the Lasagna plot is superior to use of the stacked bar chart.*

   Thank you very much for the reference, we were not aware of this manuscript. Indeed, upon closer inspection, we can confirm that our 'timesorted' lasagna plot for 1 subject is very closely related to a specific type of stacked bar chart discussed in Rodbard (2009), that is the stacked bar chart of glucose distributions as a function of time during the 24hour period (Figure 2(B) in Rodbard (2009)). Similarly, our 'daysorted' lasagna plot for 1 subject is closely related to the stacked bar chart of glucose distributions by date (Figure 2(D) in Rodbard). Nevertheless, there are several important differences.

   First, the main difference is that the stacked bar charts by definition split the glucose values into a fixed number of categories, where the same color is used within each category regardless of the value. For example, if green is used for in-range $[70, 180]$ mg/dL, then the stacked bar chart will have the same green color for values close to 70, and values close to 180. In contrast, the lasagna plots use a gradient fill, that is while a certain color is assigned to the category, the gradient of that color changes continuously with the change in glucose values. Our rationale for using a gradient fill is to soften the transition on the cutoffs, e.g. values of 179 mg/dL and 181 mg/dL are only 2 mg/dL apart. While the fixed categorical colors will change abruptly between the two, the gradient fill emphasizes that those values are indeed quite close despite being on the different sides of the cutoff. We believe this provides more detailed information on the subject's glucose profile. As an example, consider the lasagna plot for Subject 1 in the newly added red-orange color scheme in Figure R.3. On 2015-06-12, the subject had a spike in glucose values around 9am as evident by the yellow band in that time frame. Note, however, that the surrounding green is a mix between green and yellow rather than solid green, which indicates that there was a raise in glucose values that culminated in that yellow peak, followed by some decline. It is, however, possible to modify our current functionality to allow for fixed categorical colors rather than the gradient fill (which will then allow to match exactly the type of display in Figure 2(B) in Rodbard), and we hope to add this functionality in the future.

   The second difference is that the stacked bar charts in Rodbard (2009) are by definition created for one subject at a time, and are always sorted (that is they show the distribution of glucose values within the specified time range). Our lasagna plots have extended functionality in comparison. For one subject, the lasagna plots also allow the display of original glucose values by color by day without sorting (thus providing an alternative to the daily time-series plot) (Figure R.3). We believe this is useful for visual assessment of day to day variability in glucose profiles. Secondly, our package allows one to display lasagna plots for multiple subjects simultaneously, which in turn allows one to compare the average (across days) 24 hours subject profiles, or assess population-level trends across study groups. As an example, creating a lasagna plot for 5 subjects from the example dataset with 'average' datatype,

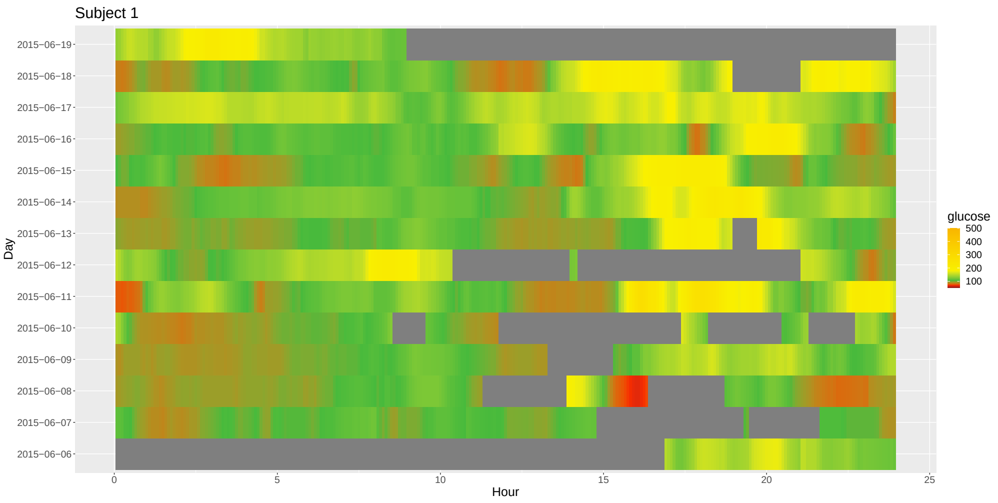

Figure R.3: Unsorted lasagna plot for Subject 1 in 'red-orange' color-scheme.

and 'subjectsorted' visually shows that Subject 2 has the highest levels of hyperglycemia, whereas Subjects 1 and 4 have the lowest levels of hyperglycemia (Figure R.4 (A)). Using the same lasagna type with 'timesorted' option instead shows that among these 5 subjects, hyperglycemia is most common in the later afternoon/evening, with the times around 4pm (16:00) and 9pm (21:00) showing the highest picks (Figure R.4 (B)). Of course, the latter conclusion is limited as it is only based on 5 subjects, however we believe creating such plots for larger subgroups may help illuminate population-level trends.

Finally, while Rodbard (2009) discussed several different types of stacked bar charts, we found that only the simplest one (a stacked bar chart for 1 subject across all times and days) is consistently used in AGP. Our new extended functionality allows one to generate such a stacked bar chart as part of the newly added AGP report (see Figure R.5 and our response to your point 4 below), or as a separate output using the function plot_ranges.

Given the discussed differences and similarities between lasagna plots and stacked bar charts, the revised manuscript provides a brief summary of this comparison:

> While lasagna plots are very similar to stacked bar charts introduced in [26] (Rodbard, 2009), there are two main differences. First, the stacked bar charts split the glucose values into a fixed number of categories (based on specified glucose cutoffs), where the same color is used within each category. In contrast, the lasagna plots use gradient fill, thus the gradient of the color changes continuously with the change in glucose values. We believe this provides more detailed information on the subject's glucose profile. Secondly, the stacked bar charts in [26](Rodbard, 2009) are created for one subject at a time. In contrast, iglu allows one to create lasagna plots for multiple subjects at once. Using datatype='average with lasagnatype = 'subjectsorted' facilitates direct cross-comparison of glucose distributions across subjects, whereas lasagnatype = 'timesorted' facilitates assessment of population-level trends. Fig R.4 shows both types of plots. Fig R.4 **A** shows that Subject 2 has the highest levels of hyperglycemia, whereas Subjects 1 and 4 have the lowest levels of hyperglycemia. Fig R.4 **B** shows that among the 5 subjects, hyperglycemia is most common in the later afternoon, with the times around 4pm (16:00) and 9pm (21:00) showing the

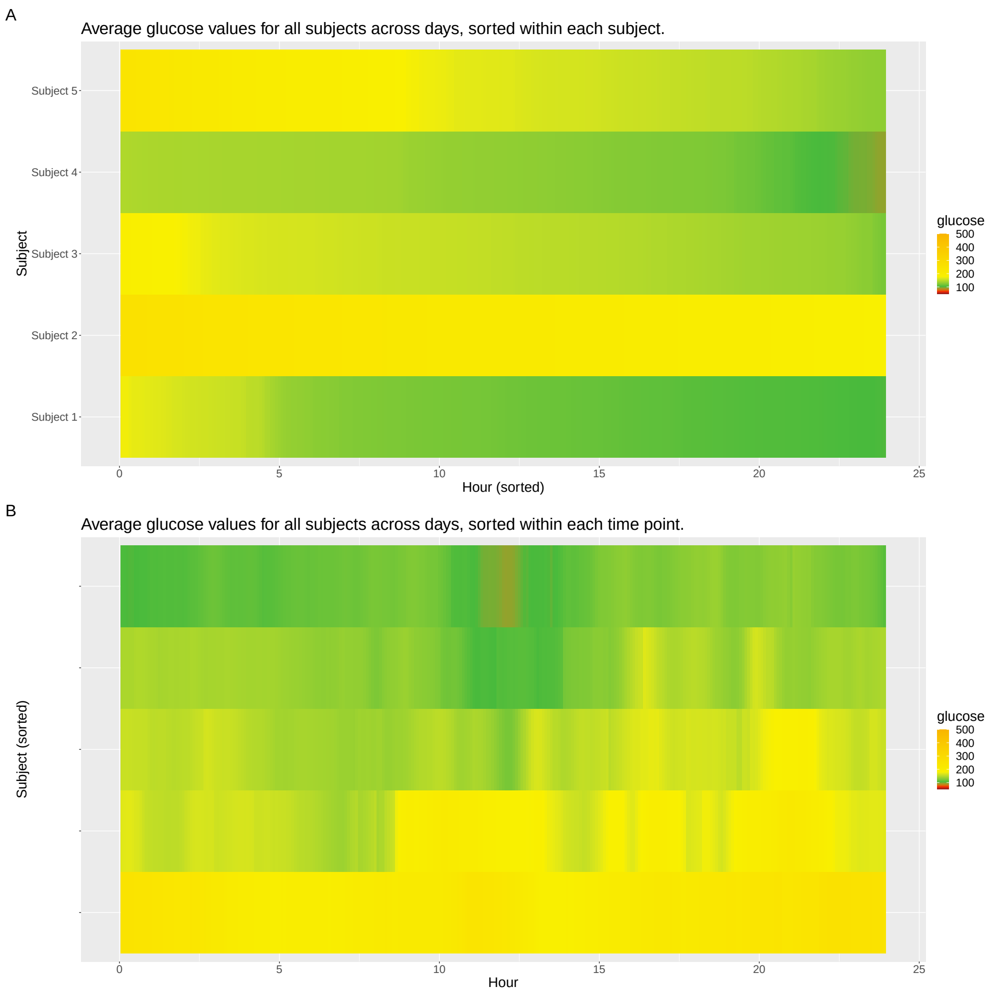

Figure R.4: Lasagna plots for 5 subjects in example dataset with 'average' datatype in 'red-orange' color-scheme. **(A)**: sorted within each subject; **(B)**: sorted within each time point across subjects.

highest glucose values.

3. *The authors note that they might add additional metrics in the future. New metrics continue to be developed. See for instance Leelarathna et al, re COGI, published in 2020 in Diabetes Technology and Therapeutics.*

   Thank you for alerting us to this new metric. The expanded package functionality now includes COGI both within R interface, and as part of the Shiny app. We updated Tables 1 and 2 accordingly.

4. *The authors do not include an AGP in their outputs. This is perhaps the most popular component of nearly all displays of CGM data at the present time – along with the stacked bar chart. See Mazze et al, Diabetes Care 1987 and also 2008 2009 and multiple other papers, and subsequent papers from Bergenstal et al. See a recent review from Rodbard in press and online at DTT.*

   Thank you for the suggestion, and the references. Indeed, the existing package functionality allowed one to calculate all standard metrics included in AGP, however we did not present

them as a separate standardized output. We have expanded the functionality of the package and the Shiny App correspondingly, both now allow the user to produce AGP output very similar to the commercial software (in line with Figure 1 in Johnson et al. 2019).

- Johnson, M. L., Martens, T. W., Criego, A. B., Carlson, A. L., Simonson, G. D., Bergenstal, R. M. (2019). Utilizing the ambulatory glucose profile to standardize and implement continuous glucose monitoring in clinical practice. Diabetes technology & therapeutics, 21(S2), S2-17.

The example AGP output from iglu is shown in Figure R.5. The output can be obtained by calling the agp function in the iglu package, or by using the new AGP tab in the Shiny App. We have added the agp function to the summary of iglu visualization capabilities in Table 3 together with the following paragraph:

> Finally, iglu allows one to generate an Ambulatory Glucose Profile (AGP) report in accordance with recommendations in [23] (Johnson et al., 2019). Figure R.5 shows an example report for Subject 1, which includes information on data collection period, time spent in standardized glycemic ranges (cutoffs of 54, 70, 180 and 250 mg/dL) displayed as a stacked bar chart [26] (Rodbard, 2009), glucose variability as measured by %CV, and visualization of quantiles of the glucose profile across days together with daily glucose views.

We have also carefully read the recommendations for improvements described in the recent review by Rodbard in DTT, and hope to implement some of these recommendations in the future (in particular adjustment of time scale to different 24 hour periods, and merging CGM data with actigraphy data).

5. *Most endocrinologists and other diabetes specialists use 70 and 180 as the cutoffs for in range. The present authors use 70 and 140. The graphs will look more familiar and appropriate to many readers if you use 70 and 180, or even 54 70 180 and 250. See Battelino et al, 2019, Diabetes Care. (63 and 140 are used or proposed for pregnancy).*

   Thank you for your comments and feedback, and for pointing us to Battelino et al., 2019. We have changed the default range in plot_glu function to $[70, 180]$ mg/dL, and have updated Figure 1 and the manuscript accordingly. We also changed the default ranges in in_range_percent function to $[70, 180]$ and $[63, 140]$, and added corresponding references to Battelino et al. (2019). The functions above_percent and below_percent by default produce output for 140, 180, 250 mg/dL and 54, 70 mg/dL, correspondingly.

6. *It is reasonable for the authors to use their own nomenclature for their own variables in their program. However, for clarity, the authors might use the nomenclature as established in the literature for a number of variables, e.g. the subclassification of SD, i.e. SDT SDw SDb SDhh:mm SDdm SDb//dm and some others. e.g., bottom of page 4/9*

   Thank you for the suggestion. In iglu software documentation files for each function and in text, we try to adhere as much as possible to the established nomenclature. We have also edited the names of the metrics displayed in the heatmap (current Figure 5), and followed the suggested nomenclature for the subclassification of SD.

   However, it is harder to control nomenclature with R specific outputs as there are restrictions on the possible variable names and column names in R. Thus, the output of sd_measures uses

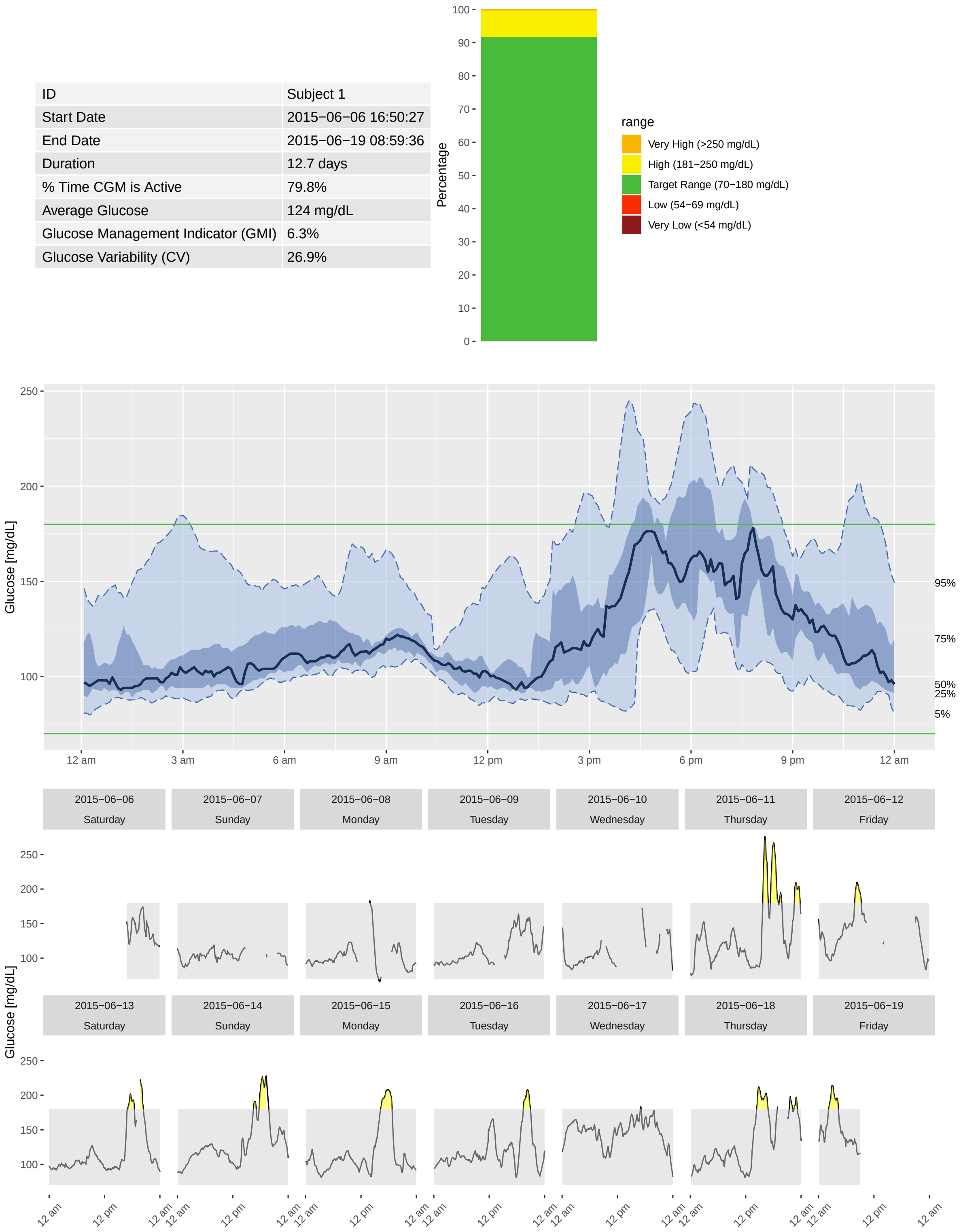

Figure R.5: Ambulatory Glucose Profile (AGP) for Subject 1 generated by iglu.

our own somewhat modified nomenclature (in particular we avoid the use of special symbols like space ‘ ‘, ‘:‘ and ‘\\‘). Nevertheless, we made slight adjustments to R outputs during revisions to hopefully better match the established notations from the literature.

Old output:

```
sd_measures(example_data_5_subject)
# A tibble: 1 x 7
  id          SdW SdHHMM SdWSH  SdDM   SdB SdBDM
  <fct>     <dbl>  <dbl> <dbl> <dbl> <dbl> <dbl>
1 Subject 1  26.4   19.6  6.54  16.7  27.9  24.0
2 Subject 2  36.7   22.8  7.62  52.0  48.0  35.9
3 Subject 3  42.9   14.4  9.51  12.4  42.8  42.5
4 Subject 4  24.5   12.9  6.72  16.9  25.5  22.0
5 Subject 5  50.0   29.6 12.8   23.3  50.3  45.9
```

New revised output:

```
sd_measures(example_data_5_subject)
# A tibble: 5 x 7
  id          SDw SDhhmm SDwsh  SDdm   SDb SDbdm
  <fct>     <dbl>  <dbl> <dbl> <dbl> <dbl> <dbl>
1 Subject 1  26.4   19.6  6.54  16.7  27.9  24.0
2 Subject 2  36.7   22.8  7.62  52.0  48.0  35.9
3 Subject 3  42.9   14.4  9.51  12.4  42.8  42.5
4 Subject 4  24.5   12.9  6.72  16.9  25.5  22.0
5 Subject 5  50.0   29.6 12.8   23.3  50.3  45.9
```

The sd_measures documentation accessible from R console by typing ?sd_measures after loading iglu now also clarifies that for example SDhhmm is known as SDhh:mm and SDbdm is known as SDb // dm. We hope that these changes improved the clarity.

7. *Several of the metrics have ‘parameters’, i.e. TIR, TBR TAR MR CONGAn IGC (with a,b,c,d, LLTR ULTR). Please specify what default parameters you are using, and whether or not the end user can adjust those parameters. Cf top of page 4/9. Also SDwsh – what are the parameters for h? and is this for the entire series with all possible starting points, or for particular starting points?*

   Thank you for your comment. Indeed, most of the parameters can be adjusted by the user, and the detailed description of each parameter together with their default values is provided within R package iglu documentation associated with each function. This documentation can be accessed directly from the R console by using help as with all other R packages, e.g. ?igc provides a description of the meaning of a, b, c, d, LLTR, ULTR in IGC metric calculations, together with all the corresponding default parameters. We acknowledge that the Shiny app is more limited in this regard as its development is a little bit behind the full R package functionality due to the difficulties associated with connecting built-in R package documentation to the app. For IGC metric, the Shiny App currently only works with default values of a, b, c, d, LLTR, ULTR (currently set as $a = 1.1$, $b = 2$, $c = d = 30$, LLTR = 80, ULTR = 140 matching the values suggested in Rodbard(2009)), however this gap will be eliminated in future updates. The full documentation for each function can be accessed from the package website https://irinagain.github.io/iglu/reference/index.html which does not require R use. As these detailed and sometimes quite technical descriptions of metric

calculations are already part of mandatory R package documentation, we have opted out of including full details in the manuscript, and instead focus on the general package overview.

In the revised version, we have made several changes to make this documentation more accessible. First, we made changes to the Shiny app so that instead of the previous generic message "Specify parameter", metric-specific parameter information is now displayed. Secondly, we clarified that the full technical details of each metric implementation together with the description of user-adjustable parameters is available in accompanied documentation, and can also be accessed directly from the website. The revised manuscript now reads

> Table 1 summarizes all the metrics implemented in the package, which can be divided into two categories: time-independent and time-dependent. All the functions assume that the glucose value are given in mg/dL units. Each function has detailed documentation that describes all the input parameters (and their default values) as well as the specific algorithm used for metric calculation. Full documentation can be accessed from the R console after loading iglu package (e.g. ? active_percent) or from the accompanying website (https://irinagain.github.io/iglu/).

We hope this makes it more clear how to use the package.

Finally, regarding your question on SDwsh, we use all possible starting points. That is, we calculate it by taking the hour-long intervals starting at every point in the interpolated grid from CGM2DayByDay (the grid matches CGM frequency, e.g. 5 min for Dexcom), computing the standard deviation of the points in each hour-long interval, and then finding the mean of those standard deviations. That is, for n time points in the grid, we compute $SD_1 ... SD_n$, where SD_i is the standard deviation of glucose values $[X_i, X_{i+1}, ... X_{i+k}]$, with the value of k (number of measurements in an hour long interval) being dependent on the meter frequency. The final number is obtained by averaging all this standard deviations, that is by taking $1/n * \sum[(SD_i)]$. This description of the implemented algorithm is available from iglu package documentation as described above.

8. *Throughout - this reviewer prefers use of 'allows one to map' rather than 'allow to map' (several other instances for the word allows)*

   Thank you for the suggestion. We have revised accordingly.

9. *Page 5/9: under visualizations, line 5: reference to Section ___ (the number of the section is not currently specified)*

   Thank you for pointing out these inconsistencies. This was an artifact of using a different latex template with numbered sections. We have revised the manuscript to omit the need for section numbers.

10. *The color scheme used by the authors is perfectly acceptable – but most of the commercial software for CGM data analysis uses a different color scheme. Authors might to well to build in flexibility and allow the end user to select their own color scheme.*

    Thank you for the suggestion. The newly included AGP output (see Figure R.5 and our response to your point 4) uses the same color scheme as the commercial software. We also added a new color_scheme parameter to lasagna plots, which allows the user to switch from the default 'blue-red' colors to the more commonly used 'red-orange' colors in stacked bar charts. Figure R.6 shows the same lasagna plot for subject 1 in both color schemes. The

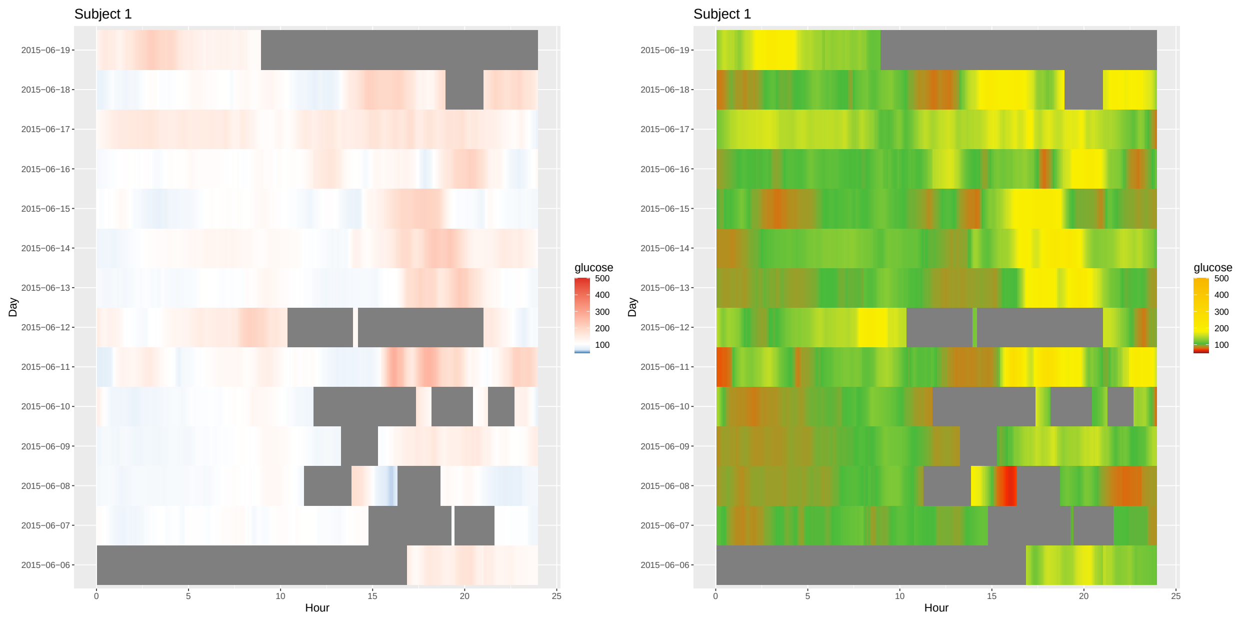

Figure R.6: Lasagna plot for Subject 1 generated by iglu with two different color schemes.

Shiny app has also been updated accordingly, where now the user has a choice between the two color schemes for lasagna plots with a clickable interface.

11. *Consider the possible option to include a logarithmic scale for glucose (Rodbard 2009 .. separate article than the ones cited).*

    Thank you for the suggestion. We have followed it, and included a new parameter log to the plot_glu function. By default, log=FALSE, leading to a linear scale. Changing log=TRUE leads to a semilogarithmic scale mimicking Figure 1(C) in Rodbard (2009):

    - Rodbard D (2009). A semilogarithmic scale for glucose provides a balanced view of hyperglycemia and hypoglycemia. J Diabetes Sci Technol. 3(6), 1395-401.

    Figure R.7 shows the glucose profile for Subject 1 from example data generated by iglu with log=TRUE. Here the glucose values are displayed in original mg/dL units, but the spacing of the values on y axis follows $\log_{10}$ transformation. The Shiny app has corresponding updates as well, where the user can now select the transformation type (none or $\log_{10}$) for plotting.

12. *Page 6/9: Authors discuss "worse glucose control" at at least two locations in the paper. But worse has several dimensions – can be in terms of TIR TBR TAR SD CV etc. So authors should be careful in using the term "worse" – it is "worse" in only 1 dimension.*

    Thank you for your comment. We have edited the writing to clarify what we mean. Specifically, we clarified that Subject 2 has the worst hyperglycemia (highest values for CGM-metrics in hyperglycemia group (2) based on hierarchical clustering in Figure 5), and Subject 5 has the worst glucose variability (highest values for CGM-metrics that measure glucose variability, group (6) based on hierarchical clustering in Figure 5).

13. *Authors seem unaware of two articles by Fabris C et al (with Breton or Cobelli or Kovatchev) using principal components analysis and the high degree of correlation of many of the metrics. These probably should be cited, vis a vis the hierarchical analysis used by the present authors.*

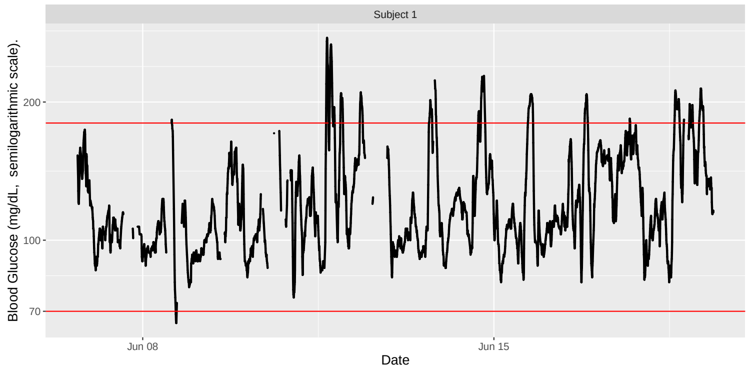

Figure R.7: Time series plot for Subject 1 using a logarithmic scale for glucose (log=TRUE in plot_glu). Selected target range is $[70, 180]$ mg/dL.

Thank you for the references, we indeed were not aware of these articles. However, investigating the general relationship between the metrics is not our main goal in this work, and is beyond the scope of this manuscript. The reason we added this section is because in the process of writing this manuscript, we realized that the readers who are new to CGM literature (in particular, statisticians who are new to this type of data) may feel overwhelmed by the sheer amount of available metrics and confused by their names, and thus may benefit from at least some guidance on the metrics concordance with each other. So our goal here is a simple illustration of metrics “closeness” on the example dataset, and we believe it is a very useful starting point for such new users.

We do, however, have an interest in further investigating the relationship between the metrics based on hierarchical clustering on a larger scale (e.g. using data from subjects with both Type I and Type II diabetes across several CGM meters with larger sample sizes), and comparing our approach and conclusions with the work of Fabris C et al. In particular, the latter have specifically focused on glucose variability metrics (in contrast to all the metrics), and in Fabris et al. (2016) the authors indeed indicate that it would be of interest to expand their metrics list. From the statistical standpoint, the work of Fabris et al. is also quite different in that they focus more on a new composite measures (via SPCA) based on a selected set of variables, whereas we focus on exploratory analysis by clustering all metrics into interpretable groups. That is, we do not aim to select a smaller set of metrics, rather we aim to give some guidance on which metrics could be grouped together in a data-driven fashion. We added the references with the following contrast in the manuscript:

> The relationship between a reduced list of metrics has also been studied in [29,30] (Fabris et al., 2014, 2015) using sparse principal component analysis. While [29,30] focus on selection of a few key metrics to describe glucose variability, our goal here is exploratory analysis to illustrate differences and similarities between all metrics on a given dataset.

Indeed, we are very thankful for pointing out this work, and we are very much inspired to investigate the relationship between the metrics further and expand on the work of Fabris et al. We aim to pursue these questions in future work as it’s outside of the scope of this manuscript.

14. *What algorithm are the authors using for MAGE (please use MAGE in all caps – all uppercase). Peter Baghurst has an algorithm (DTT) written in R. Are you using that one? That has been 'validated' or at least tested – see Sechterberger et al.*

    Our original reference for all the CGM metrics is Rodbard (2009), thus in calculating MAGE we followed the description provided there. Specifically, we take the mean of absolute differences (between each glucose value and the glucose mean) that are greater than one standard deviation (an optional sd_multiplier parameter can be used to adjust e.g. two standard deviations instead of one). These details of MAGE calculation are part of iglu software documentation, and can be obtained from CRAN (https://cran.rstudio.com/web/packages/iglu/iglu.pdf), the package website (https://irinagain.github.io/iglu/reference/mage.html) or by accessing help for the corresponding function from the R console (? mage). In figures, Shiny App, in text and in documentation we always refer to MAGE as all caps. However, for consistency of R function names for metrics and R coding style, we prefer to keep all metric function names lower case.

    We agree that the currently used MAGE algorithm is likely not the most accurate as it does not identify peaks and nadirs. We are familiar with Baghurst's work in DTT, however we were not able to find an original R implementation of their algorithm. We found that cgmanalysis package in R calculates MAGE based on Baghurst's algorithm, however some choices in implementation are still somewhat subjective (e.g. the use of peak/nadir identification algorithm after smoothing). In fact, while we were researching various algorithms for MAGE calculation in the literature to determine which one to adopt for iglu, we have developed our own algorithm for MAGE that we believe to be more accurate based on cross-comparison of automatic (via algorithm) and manual MAGE calculations (as per Service) of multiple CGM profiles. This work is not yet complete as we are in the process of finalizing the algorithm's validation and the corresponding manuscript, but once the work is complete, we plan to include this new algorithm in iglu.

15. *CVsd is an entirely new metric—what does it mean? What is it correlated with? Any place where the authors believe it might be helpful?*

    The main goals of this work are to provide researchers with (i) comprehensive software for evaluation of various CGM metrics that is (ii) easy to use. Thus, it is not our goal here to give recommendations on which metrics should be used in practice for assessment of various clinical outcomes, nor do we aim to make any claims on one metric's superiority over the other. While we agree that CVsd is not as clearly defined in the literature as some other metrics, there is undeniably a lot of interest in measuring both within-day (intra-day) and between-day (inter-day) glucose variability. It is our opinion, however, that the literature (e.g. Umpierrez at al., 2018, Am J Med Sci) tends to be vague on how exactly one may do so if using CV as a measure rather than SD.

    With this in mind, our rationale for implementing CVsd was two-fold.

    First, we wanted to implement a glucose varaibility measure based on CV that differentiates within-day (intra-day) and day-to-day (inter-day) variations. While CVmean can be interpreted as a measure of **average intra-day variation**, CVsd allows one to measure **inter-day variations in glucose variability** itself. Our hierarchical clustering results do indeed indicate that while CVsd is correlated with other variability metrics (e.g. CV and CVmean) more so than it is with average metrics (e.g. Mean and Median), CVsd is also quite different due to its focus on **inter-day variations in glucose variability** rather than variability itself, although of course this analysis is limited to 5 subjects. In the example

dataset, CVsd is largest for Subject 3 (recall that Subject 2 has the highest proportion of time above 180 mg/dL, and Subject 5 has the highest glucose variability as measured by SD). The reason CVsd is highest for Subject 3 is because that subject has highest variability changes from day to day, that is on some days its CV is quite low, and on some days its CV is very high. In contrast, Subject 5 has consistently high variability (its CV remains high on all days leading to higher CVmean but lower CVsd).

```
cv_measures(example_data_5_subject)
# A tibble: 5 x 3
  id        CVmean  CVsd
  <fct>      <dbl> <dbl>
1 Subject 1   21.1  7.80
2 Subject 2   17.0  6.41
3 Subject 3   27.1  9.40
4 Subject 4   18.4  5.70
5 Subject 5   29.0  7.56
```

There could be several explanations for this, perhaps Subject 3 has larger inconsistencies across days (e.g. in terms of meal composition and exercise), but we refrain from further analyses here as this is not our main goal.

Our 2nd reason for implementing CVsd is for convenience of follow-up statistical analyses. Specially, CVsd allows one to assess uncertainty in the values of CVmean for each subject (by e.g. allowing one to construct confidence intervals for average intra-day CV based on CVmean, CVsd and # of available days as returned by active_percent function). We focus on returning this measure for CV specifically rather than all variability metrics as we found CV to be the most commonly reported metric.

We are thankful for you framing these questions on clinical utility of CVsd as they inspired us to have a follow up work on this topic.

16. *In your function CGMS2DaybyDay, what is the nature of your smoothing function? Any particular reason why it was selected or what its advantages might be relative to some alternatives? Any indication that it performs better than others.*

    Thank you for your comment. The purpose of CGMS2DayByDay is not to perform smoothing per se, but rather to put the glucose values on a uniform time grid within a 24 hour period across days and subjects. We found this necessary to be able to efficiently calculate multiple glucose variability metrics (e.g. CONGA and subtypes of standard deviation from Rodbard (2009) that work on equidistant time grid), and we wanted to ensure that the same grid is used across all subjects. Suppose the original measurements are collected at 15:31, 15:36, 15:41, 15:46, etc for Subject 1, and at 15:33, 15:38, 15:43, 15:48 for Subject 2. Then the function returns values at 15:30, 15:35, 15:40, 15:45 for both subjects, which are obtained by linearly interpolating their respective glucose values at neighboring time points. Visually, we found such a linearly interpolated CGM profile indistinguishable from the original profile.

    While not the original goal, such linear interpolation does allow us to perform some smoothing by replacing missing values with interpolated values between observed measurements that are less than inter_gap minutes (by default we use a gap of 45 minutes, but this can be adjusted by the user). Our rationale for the inter_gap parameter is as follows. Suppose the meter frequency is 5 minutes, and we observe values 100, NA, 150 at time points 0, 5 and 10

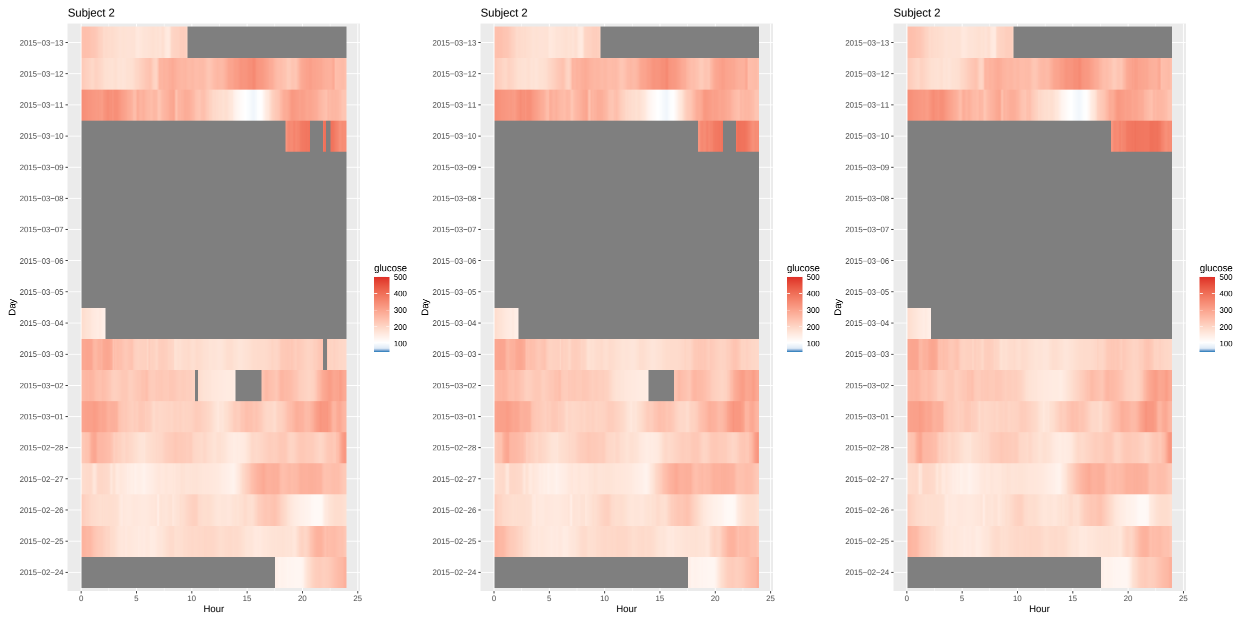

Figure R.8: Unsorted lasagna plots for Subject 2 using different values of inter_gap parameter for linear interpolation: from left to right the values of 10 min, 45 min (default), and 150 min (2 hours, 30 min) are used, correspondingly.

minutes. Given that glucose values cannot biologically change too abruptly, we believe it is reasonable in this case to interpolate the NA value at 5 min, and replace that NA value with 125. On the other hand, for Subject 2 (see Figure R.2) there is a large gap of measurements due to missing values for several days, and we believe it's impossible to accurately impute those values. Thus, applying the CGMS2DayByDay() function to Subject 2 data will leave that large gap as NA. To better illustrate the effect of the inter_gap parameter, Figure R.8 shows lasagna plots for Subject 2 with various values for inter_gap. Observe that the large interval of missing data consistently remains NA, whereas the smaller intervals get filled in as the value of inter_gap grows.

We have added additional clarifications on CGMS2DayByDay() in the manuscript which now reads as follows:

> The calculations of these variability metrics require evenly spaced glucose measurements across time; however, this is not always the case in practice due to missing values and misalignment of CGM measurement times across subjects (e.g. measurement at 17:30 for Subject 1, but at 17:31 for Subject 2). In order to create a uniform evenly spaced grid of glucose measurements, iglu provides the function CGMS2DayByDay. This function is automatically called for metrics requiring the evenly spaced grid across days, however the user can also access the function directly. The function works on a single subject's data, and has three outputs.

```
str(CGMS2DayByDay(example_data_1_subject))
List of 3
  $ gd2d        : num [1:14, 1:288] NA 112.2 92 90.1 143.1 ...
  $ actual_dates: Date[1:14], format: "2015-06-06" "2015-06-07" ...
  $ dt0         : num 5
```

> The first part of the output, gd2d, is the interpolated grid of values. Each row corresponds to one day of measurements, and the columns correspond to an equi-distant time grid covering a 24 hour time span. The grid is chosen to match the frequency of the sensor (5 minutes in this example leading to $(24 * 60)/5 = 288$ columns), which is returned as dt0. The linear interpolation is only performed between observed CGM values that are less than inter_gap minutes apart, otherwise missing values are inserted. By default, the function uses inter_gap = 45 minutes, however this value can be adjusted by the user. The returned actual_dates allows one to map the rows in gd2d back to the original dates. The achieved alignment of glucose measurement times across the days enables both the calculation of corresponding metrics, and the creation of lasagna plots discussed in the next section.

We do, however, think it is worthwhile to investigate smoothing on its own to reduce measurement error, and to have a more principled interpolation approach. It is, however, not clear to us yet what is the best smoothing method to utilize for CGM data, and how much it will affect the reliability of resulting estimates. Thus, for simplicity, currently iglu works with non-smoothed original CGM measurements. While the linear interpolation in CGMS2DayByDay() does some alteration of original data, we argue that it is both quite minor given visually indistinguishable profiles, and is an easy processing step to explain to a wide audience (compared to, say, smoothing via functional principal component analysis which we did for average CGM trajectories during sleep in Gaynanova I, Punjabi N, Crainiceanu C. Modeling continuous glucose monitoring (CGM) data during sleep. Biostatistics. DOI: https://doi.org/10.1093/biostatistics/kxaa023).

We plan to investigate various smoothing options in future work, and hope to add such additional smoothing capabilities to iglu in the future.

17. *You do not seem to include MAG (DeVries, Hermanides ?) also also see distance traveled (DT from Marling). A recent study from Moscada (?) and Nick Oliver indicates that MAG has the highest value for Discriminant Ratio, a criterion they propose as a basis to select among alternative metrics for various aspects of CGM.*

    Thank you for the suggestion. We have updated the functionality of both the package and the Shiny app to include MAG. Tables 1 and 2 have also been updated accordingly. We have not included distance traveled (DT) at this time, but hope to include it in the future.

18. *Various consensus papers (Bergenstal, Danne, Maahs, Battelino) have discussed the statistics that the consensus groups believed should be presented together with the AGP (and other analyses of the CGM data). Please be sure that you have computed and provide the ones that are currently or recently in vogue.*

    Thank you very much for the suggestion and references. Indeed, almost all of the metrics from consensus papers (e.g. time in ranges, LBGI/HBGI, AUC, mean, SD, CV, estimated A1C, GMI) can be calculated with iglu, however they were not presented as part of the standardized output. In the revised version, iglu is now able to produce a standardized AGP output in line with recent recommendations in Johnson et al., 2019 as shown in Figure R.5 (see also our response to your point 4). This includes information on data collection (date range, % of time CGM is active), average glucose value (as measured by mean), estimated A1C (as measured by GMI), glucose variability (as measured by % CV), % of time spent in pre-specified cutoff ranges (54, 70, 180, 250 mg/dL) displayed in a stacked bar chart, quantiles corresponding to a specific time of day, and daily profiles with shaded areas for values < 70 mg/dL (shaded in

red), and > 180 mg/dL (shaded in yellow). After studying the consensus papers carefully, we found that the only missing functionalities are (i) separation of output into wake and sleep periods based on pre-determined time ranges; (ii) count of the number of hypoglycemia and hyperglycemia episodes; (iii) glucose stability as measured by the mean hourly change from the median curve (Bergenstal et al., 2013). We hope to expand the functionality even further in the future to add these metrics.

19. *Several authors have investigated how much data must be available to obtain reliable estimates of the parameters. These include Xing et al, Riddlesworth et al, and Nick Oliver or others from his group (may be in press or online at the present time, in DTT).*

    Thank you for the comment. We are indeed aware of Xing et al and Riddlesworth et al recommendations of 10-14 days of CGM measurements, however the prior functionality of iglu did not produce a clear output for the user on data availability. In the revised version, this information is included in the new standardized AGP report (see Figure R.5 and our response to your point 4), and can also be obtained by calling the active_percent function from within R, or choosing the "Active Percent" metric from the Shiny app. We have added explicit description of the active_percent function to the manuscript together with a summary of recommendations from Riddleswoth et al. 2018 based on their conclusions.

    > Finally, iglu also allows one to assess the reliability of estimated CGM metrics by providing information on the number of days of data collection together with % of time the CGM device was active during those days (% of non-missing measurements). This information is automatically provided as part of the standardized AGP output discussed in the next section, and can also be obtained directly by calling the function active_percent.
    >
    > ```
    > active_percent(example_data_5_subject)
    > # A tibble: 5 x 5
    >   id        active_percent ndays     start_date          end_date
    >   <fct>              <dbl> <drtn>    <dttm>              <dttm>
    > 1 Subject 1           79.8 12.7 days 2015-06-06 16:50:27 2015-06-19 08:59:36
    > 2 Subject 2           58.9 16.7 days 2015-02-24 17:31:29 2015-03-13 09:38:01
    > 3 Subject 3           92.1  5.8 days 2015-03-10 15:36:26 2015-03-16 10:11:05
    > 4 Subject 4           98.7 12.9 days 2015-03-13 12:44:09 2015-03-26 10:01:58
    > 5 Subject 5           95.8 10.6 days 2015-02-28 17:40:06 2015-03-11 08:04:28
    > ```
    >
    > According to [17] (Riddlesworth et al., 2018), 10-14 days of CGM measurements are generally sufficient for assessing outcomes in clinical trials, and for determining potential adjustments to diabetes management based on retrospective review. Given these recommendations, the estimates of CGM parameters for Subject 3 are less reliable than the estimates for other subjects in the example dataset.

20. *There are two major sets of applications for CGM data: The first is for the care of the individual patient. For this, one does not need or want all possible parameters... there is now a consensus that TIR TAR TBR Mean or median, %CV, and perhaps a very few other parameters are sufficient – in any event they are usually more than either the physician, health care practitioner, patient or patient's family can digest. So, there is very little demand for new metrics – most unfamiliar, and most highly redundant or at least highly correlated with the few mentioned here.*

    We agree that for clinical practice, the availability of additional CGM measures that are likely to be correlated with other commonly available measures (e.g., TIR, TAR, etc.) will not have

significant utility in day to day practice. We, however, respectfully disagree that, in the context of research, these new measures are of potential value. CGM measures provide dynamic characterization of glucose trajectories which can be of immense value when considering the potential impact of conditions that are associated with acute temporal changes in pathophysiological mechanisms that can impact glucose homeostasis. For example, sleep apnea is a common condition that affects 9% of women and 25% of men in the general population. It is well known that sleep apnea is associated with nocturnal repetitive increases in sympathetic activity due to cyclical hypoxemia and recurrent arousals from sleep. Thus, to determine whether these acute changes which are known to increase sympathetic nervous system activity can influence glucose homeostasis, measures that capture the dynamic nature of glucose trajectories are needed. Even in clinical scenarios where acute pathophysiological changes are not present, metrics that help probe the temporal nature of glucose are of value. For example, obesity is associated with metabolic flexibility. Having detailed CGM measures that help define the various rates of change (increase and decrease in glucose levels) can provide insight into how conditions, such as obesity and polycystic ovary syndrome, alter the diurnal nature of glucose profiles. We have modified the conclusions section of the manuscript to acknowledge both the limited utility of the full list of available metrics for individual patient care as well as potential value of metrics that measure dynamic nature of glucose trajectories.

21. *The other major application of CGM data is in the context of clinical trials. Here one is interested in significance testing for superiority or non-inferiority for one or the other treatment, using randomized parallel studies or crossover studies. These often involve multivariate corrections e.g. for baseline A1C, or baseline mean glucose, and for clinical sites, and other subsets of the data. This is what a pharmaceutical laboratory would need. The present article, and program, despite all of its strengths, does not really fulfil the needs to these two major potential groups of users (in my opinion). The current study and paper is still valuable, and publishable, but this reviewer would suggest that the authors try to address or at least acknowledge these two major use-cases, and either try to address them in the present paper, or address them in future studies and manuscripts. Perhaps the lack of the ability of the present study and code to address these important use-cases should be included as a limitation of the study. However, the present code should be useful to others trying to address those cases.*

    We appreciate the reviewer's comments that CGM data are of value in the context of clinical trials (and thus significant testing). In fact, the work presented in the current manuscript under review was motivated by a recently completed clinical trial on the effects of sleep apnea treatment with positive airway pressure on CGM profiles. Given the detailed nature of CGM data and the increasing use of acquiring such data, we believe that convenient methods for analyzing CGM data are desperately needed to facilitate use of CGM methodology by investigators in observational studies and randomized clinical trials. We have modified the conclusions section of the manuscript accordingly.

22. *Hill and Oliver et al have recently published (or have online) a new version of their EZ GV method (still in Excel, I believe) that might be helpful.*

    Thank you for the reference, we added it to the manuscript in the Introduction.

    > EasyGV is a free CGM software in the form of a macro-enabled Excel workbook (Hill et al., 2011), and thus is more accessible compared to CGManalyzer and cgmanalysis. However, it only allows calculation of 10 metrics. Furthermore, unlike R, Excel is not a script-based programming language, which makes it less desirable

for those users who want to create reproducible scripts for all data processing and metric calculation steps. Thus, there remains a need for open-source software that (i) computes most of the CGM metrics available from the literature, and (ii) meets the needs of researchers with varying levels of programming experience.

23. *Some of the references cited in my comments above: Some of these might be helpful to the authors in the context of the present paper; others may be helpful in the future, to enable them to better coordinate with the "clinical diabetes" side of the relevant literature.*

    Thank you very much for these references. We are very grateful, and it's extremely helpful.

References:

1. Updated Software for Automated Assessment of Glucose Variability and Quality of Glycemic Control in Diabetes. Moscardó V, Giménez M, Oliver N, Hill NR.Diabetes Technol Ther. 2020 Oct;22(10):701-708. doi: 10.1089/dia.2019.0416. Epub 2020 Apr 22.PMID: 32195607

2. Assessment of Glucose Control Metrics by Discriminant Ratio. Moscardó V, Herrero P, Reddy M, Hill NR, Georgiou P, Oliver N.Diabetes Technol Ther. 2020 Oct;22(10):719-726. doi: 10.1089/dia.2019.0415.PMID: 32163723

3. Calculating the mean amplitude of glycemic excursion from continuous glucose monitoring data: an automated algorithm. Baghurst PA.Diabetes Technol Ther. 2011 Mar;13(3):296-302. doi: 10.1089/dia.2010.0090. Epub 2011 Feb 3.PMID: 21291334

4. Ambulatory glucose profile: representation of verified self-monitored blood glucose data R S Mazze, D Lucido, O Langer, K Hartmann, D Rodbard PMID: 3552508 DOI: 10.2337/diacare.10.1.111

5. Recommendations for standardizing glucose reporting and analysis to optimize clinical decision making in diabetes: the Ambulatory Glucose Profile (AGP). Bergenstal RM, Ahmann AJ, Bailey T, Beck RW, Bissen J, Buckingham B, Deeb L, Dolin RH, Garg SK, Goland R, Hirsch IB, Klonoff DC, Kruger DF, Matfin G, Mazze RS, Olson BA, Parkin C, Peters A, Powers MA, Rodriguez H, Southerland P, Strock ES, Tamborlane W, Wesley DM.Diabetes Technol Ther. 2013 Mar;15(3):198-211. doi: 10.1089/dia.2013.0051. Epub 2013 Feb 28.PMID: 23448694

6. J Diabetes Sci Technol . 2009 Nov 1;3(6):1395-401. doi: 10.1177/193229680900300620. A semilogarithmic scale for glucose provides a balanced view of hyperglycemia and hypoglycemia David Rodbard 1 PMID: 20144394 PMCID: PMC2787040 DOI: 10.1177/193229680900300620

7. Clinical Targets for Continuous Glucose Monitoring Data Interpretation: Recommendations From the International Consensus on Time in Range. Battelino T, Danne T, Bergenstal RM, Amiel SA, Beck R, Biester T, Bosi E, Buckingham BA, Cefalu WT, Close KL, Cobelli C, Dassau E, DeVries JH, Donaghue KC, Dovc K, Doyle FJ 3rd, Garg S, Grunberger G, Heller S, Heinemann L, Hirsch IB, Hovorka R, Jia W, Kordonouri O, Kovatchev B, Kowalski A, Laffel L, Levine B, Mayorov A, Mathieu C, Murphy HR, Nimri R, Nørgaard K, Parkin CG, Renard E, Rodbard D, Saboo B, Schatz D, Stoner K, Urakami T, Weinzimer SA, Phillip M.Diabetes Care. 2019 Aug;42(8):1593-1603. doi: 10.2337/dci19-0028. Epub 2019 Jun 8.PMID: 31177185 Free

8. International Consensus on Use of Continuous Glucose Monitoring. Danne T, Nimri R, Battelino T, Bergenstal RM, Close KL, DeVries JH, Garg S, Heinemann L, Hirsch I, Amiel SA, Beck R, Bosi E, Buckingham B, Cobelli C, Dassau E, Doyle FJ 3rd, Heller S, Hovorka R, Jia W, Jones T, Kordonouri O, Kovatchev B, Kowalski A, Laffel L, Maahs D, Murphy HR, Nørgaard K, Parkin CG, Renard E, Saboo B, Scharf M, Tamborlane WV, Weinzimer SA, Phillip M.Diabetes Care. 2017 Dec;40(12):1631-1640. doi: 10.2337/dc17-1600.PMID: 29162583 Free PMC article. Review

9. Outcome Measures for Artificial Pancreas Clinical Trials: A Consensus Report. Maahs DM, Buckingham BA, Castle JR, Cinar A, Damiano ER, Dassau E, DeVries JH, Doyle FJ 3rd, Griffen SC, Haidar A, Heinemann L, Hovorka R, Jones TW, Kollman C, Kovatchev B, Levy BL, Nimri R, O'Neal DN, Philip M, Renard E, Russell SJ, Weinzimer SA, Zisser H, Lum JW.Diabetes Care. 2016 Jul;39(7):1175-9. doi: 10.2337/dc15-2716.PMID: 27330126 Free PMC article. Review

10. Evaluating Glucose Control With a Novel Composite Continuous Glucose Monitoring Index. Leelarathna L, Thabit H, Wilinska ME, Bally L, Mader JK, Pieber TR, Benesch C, Arnolds S, Johnson T, Heinemann L, Hermanns N, Evans ML, Hovorka R.J Diabetes Sci Technol. 2020 Mar;14(2):277-283. doi: 10.1177/1932296819838525. Epub 2019 Mar 31.PMID: 30931606 Free PMC article.

11. A Review of Continuous Glucose Monitoring-Based Composite Metrics for Glycemic Control. Nguyen M, Han J, Spanakis EK, Kovatchev BP, Klonoff DC.Diabetes Technol Ther. 2020 Aug;22(8):613-622. doi: 10.1089/dia.2019.0434. Epub 2020 Mar 4.PMID: 32069094
